# Optimizing Cardiovascular Outcomes With Bisoprolol: An Evidence-Based Perspective

**DOI:** 10.7759/cureus.89579

**Published:** 2025-08-07

**Authors:** Kamal Sharma, Sunil Sathe, Bhupen Desai, Subhash Manchanda, Jagdish Mohan, Manish Bansal, Nagamalesh U. M., Abraham Oomman, Arindam Pande, Jay Shah, Johann Christopher, Sachin Patil, Jabir Abdullakutty, Akshay Bafna, Sarita Rao

**Affiliations:** 1 Cardiology, SAL Hospital, Ahmedabad, IND; 2 Cardiology, Cardiac Care and Counselling Center, Pune, IND; 3 Cardiology, Desai Heart Care Clinic, Mumbai, IND; 4 Department of Cardiology, Sir Ganga Ram Hospital, Delhi, IND; 5 Department of Cardiology, Jaipur Golden Hospital, New Delhi, IND; 6 Cardiology, Medanta The Medicity, Gurugram, IND; 7 Cardiology and Interventional Cardiology, Aster Hospital, Bangalore, IND; 8 Department of Cardiology, Apollo Hospitals, Chennai, IND; 9 Cardiology, Medica Super Specialty Hospital, Kolkata, IND; 10 Cardiology, Healthcare Global Enterprise (HCG) Multi Specialty Hospital, Ahmedabad, IND; 11 Cardiology, Care Hospitals, Hyderabad, IND; 12 Cardiology, Sachin Multispecialty Hospital, Kolhapur, IND; 13 Cardiology, Lisie Hospital, Kochi, IND; 14 Cardiology, Star Superspeciality Hospital, Kolhapur, IND; 15 Department of Cardiology, Apollo Hospitals, Indore, IND

**Keywords:** beta blocker, bisoprolol, cardiovascular disease, heart failure, hfref: heart failure with reduced ejection fraction

## Abstract

The cardiovascular continuum is the developmental process of cardiovascular diseases (CVDs) leading to heart failure (HF) and sudden cardiac death. Beta-blockers (BBs) are at the forefront of managing conditions along this continuum, ranging from cardiovascular (CV) risk factors to heart failure. In particular, bisoprolol proved to be a highly cardio-selective BB with a favourable pharmacokinetic profile, demonstrating long-term safety, good tolerability, and proven efficacy in reducing cardiac events, including arrhythmias and mortality in patients with heart failure with reduced ejection fraction (HFrEF). This evidence-based perspective showcased numerous clinical studies revealing the utility of bisoprolol in managing patients with CVDs, particularly HFrEF and stable angina. It also included safety evidence for bisoprolol in patients with renal and hepatic dysfunction, etc. Expert opinions from leading cardiologists across India further reinforce the role of bisoprolol as a first-line therapy in managing HFrEF and stable angina, making its usage suitable for HFrEF and angina patients with special emphasis on comorbidities, such as chronic kidney disease (CKD), chronic obstructive pulmonary disease (COPD), and diabetes.

## Introduction and background

Introduction

**Figure 1:**
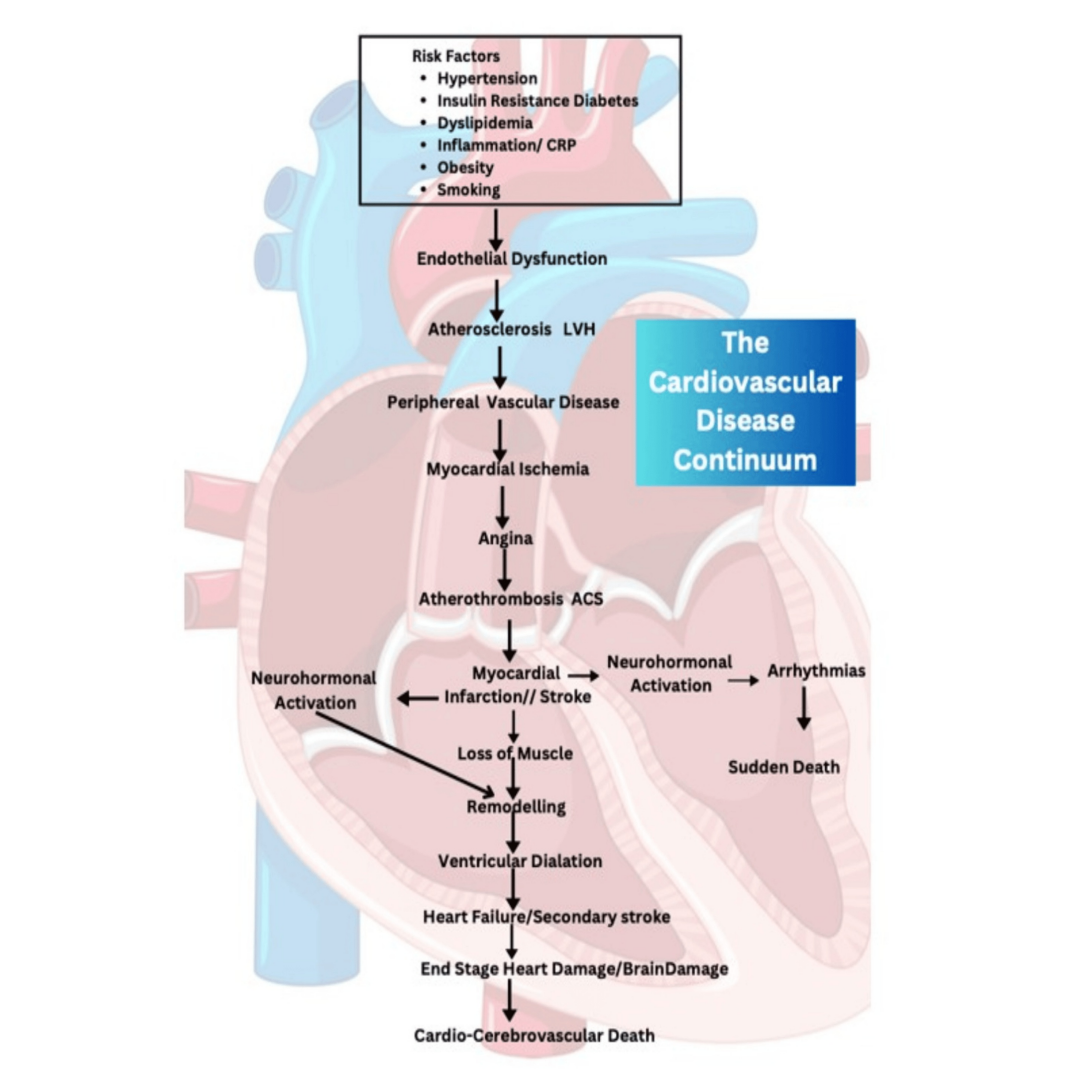
The cardiovascular continuum Reference no. [2].

The cardiovascular continuum was first described in 1991 by Dzau and Braunwald as a new strategy for the management of cardiovascular diseases (CVD) [1]. The cardiovascular disease continuum begins with risk factors for cardiovascular diseases (CVDs), chronic stable angina, myocardial infarction (MI), and asymptomatic left ventricular dysfunction, and extends to refractory heart failure (HF) and death (Figure 1) [2]. Angina pectoris and HF are among the most prevalent CVDs.

HF accounts for a large proportion of global health problems and is expected to affect approximately 64 million people worldwide [3]. In India, HF with reduced ejection fraction (HFrEF) is the most common type of HF, as shown by the Trivandrum HF registry (THFR) [4]. Similarly, the Kerala Acute Heart Failure Registry (CSI-KHFR) claims that 67.5% of patients with acute heart failure (AHF) exhibit reduced ejection fraction, primarily in younger populations [5].

Understanding the underlying pathophysiology is crucial for guiding targeted therapies. Central to the progression of HF are three neurohormonal systems: the sympathetic nervous system (SNS), renin-angiotensin-aldosterone system (RAAS), and natriuretic peptide system (NPS) [6]. These systems initially help maintain adequate cardiac output by increasing the heart rate (HR) and blood volume. However, chronic activation leads to adverse cardiac remodelling and progression to HF [7]. According to the CSI-KHFR, in patients with HF, the mortality rate is reported to be 59% at 5 years, and sudden cardiac death is the reason in 46% of the cases, showing a poor prognosis associated with this condition [5].

Another cardiovascular (CV) ailment, angina pectoris, results from an unbalanced supply-to-demand ratio of oxygen in the myocardium. Angina is not a standalone diagnosis but a symptom of the underlying cause of coronary artery disease (CAD), which can lead to myocardial ischemia and infarction. The pathophysiology of angina is multifactorial, involving epicardial coronary artery obstruction usually due to atherosclerosis, microvascular dysfunction, endothelial dysfunction, and dynamic plaque behavior [8]. In patients with angina, increased HR shortens myocardial diastole and consequently increases oxygen demand [9]. While HR suppression is considered a critical component in alleviating ischemic symptoms, addressing the broader pathophysiologic contributor is essential. Beta-blockers (BBs) by reducing myocardial oxygen demand, exert anti-ischemic effects and also provide broader cardiovascular benefits, including controlling heart rate, and improve survival in HF [10]. Both the THFR [4] and CSI-KHFR [5] registries highlight BB as one of the four cornerstone disease-modifying therapies shown to significantly improve long-term outcomes in patients with HFrEF.

Building on this rationale, bisoprolol, a cardioselective beta blocker (BB), plays a well-established role in the management of HFrEF and stable angina pectoris, primarily by reducing sympathetic overactivity and myocardial oxygen demand through inhibition of the overactive sympathetic nervous system (SNS) and renin-angiotensin-aldosterone system (RAAS). These characteristics also make bisoprolol a rational choice for long-term management of CVDs, especially in the Indian context, where comorbidities such as chronic kidney disease (CKD), diabetes, and chronic obstructive pulmonary disease (COPD) are common.

Objective of the meeting

Recognizing the evolving clinical landscape and real-world considerations of bisoprolol in cardiovascular care, this evidence-based perspective paper synthesizes insights from a forum of Indian cardiologists. The objective of this opinion paper was to provide a focused review on the clinical utility of BBs, particularly bisoprolol, not only in the later stages of the cardiovascular continuum, like HFrEF, but also in earlier phases, such as stable angina, which remains the central focus of this discussion. Additionally, the paper seeks to explore its potential application in patients with commonly encountered comorbidities, acknowledging the multifaceted nature of patient care in routine clinical practice.

## Review

Methodology

A series of structured expert group meetings were conducted with 56 leading cardiologists from across India representing all major regions-North (16), East (6), West (19) and South (15) Discussions focused on the positioning of bisoprolol among BBs in guideline-directed medical therapy (GDMT), its use in patients with comorbid conditions, and specific patient profiles favoring bisoprolol. Key discussion points included receptor selectivity, dosing considerations, and real-world applicability across diverse patient subgroups.

Beta blockers

Beta blockers (BBs) primarily act on β1 adrenergic receptors in cardiac myocytes and juxtaglomerular cells of the kidney and work by blocking β1 adrenoreceptors, leading to reduced HR and consequent reduction in myocardial oxygen demand and cardiac workload. This makes them a critical component in the management of multiple CVDs, including HFrEF and stable angina pectoris [10]. Additionally, non-selective BBs block β2 receptors, mainly found in vascular and airway smooth muscle, potentially causing bronchoconstriction and vasoconstriction, thus requiring caution in patients with asthma or peripheral artery disease [11].Bisoprolol

Bisoprolol is a cardioselective β1-adrenergic receptor antagonist and is among the GDMTs for HFrEF that are intended to reduce the mortality rate and hospital readmissions based on the 2022 AHA/ACC/HFSA guidelines [12]. It helps in reducing morbidity and mortality post-myocardial infarction (MI) and the occurrence of stroke and CAD [13]. Bisoprolol's favorable pharmacokinetic (PK) profile supports its use as a BB of choice within the GDMT framework. It is well-absorbed with 90% oral bioavailability. Maximum plasma levels are attained approximately 3 hrs after a dose of 10 mg bisoprolol [14]. Approximately 50% of bisoprolol is metabolized by the liver, mainly involving CYP3A4, and is converted into inactive metabolites, while the other 50% is excreted unchanged by the kidneys [15]. Its elimination half-life is 10-11 hours in healthy individuals, extending to 18 hours in those with renal impairment and 13 hours in individuals with hepatic cirrhosis, supporting once-daily dosing [16]. Due to its high oral bioavailability and low first-pass metabolism, bisoprolol has linear pharmacokinetics over a wide range of dosage regimens [17]. Comparison of PK properties of bisoprolol vs other BBs is mentioned in Table 1.

**Table 1:** Comparative profile of beta blockers

Parameter	Bisoprolol	Carvedilol	Metoprolol Succinate	Nebivolol
β1 Selectivity [18]	High (β1-selective) (Affinity (pK_D_) β1=7.43-7.98; β2=5.42-6.70; β3=5.04-5.67)	Non-selective (β1, β2) + α1-blocking (Affinity (pK_D_) β1=8.75-9.26; β2=8.96-10.06 β3=6.61-8.30)	Moderate (β1-selective) (Affinity (pK_D_) β1=7.26-7.36; β2=5.49-6.89; β3=5.00-5.16)	High (β1-selective) (Affinity (pK_D_) β1=8.79-9.17; β2=6.55-7.96 β3=5.66-7.04)
Vasodilatory Properties [19]	None	Yes (via α1-blockade)	None	Yes (via nitric oxide release)
Half-life (hours) [19]	9–12	7–10	3–4	10–30( depending on CYP polymorphism)
Metabolism [20]	50% hepatic, 50% renal; no dose adjustment in hepatic/renal impairment	Hepatic (CYP2D6); caution in hepatic impairment	Hepatic (CYP2D6); caution in hepatic impairment	Hepatic (CYP2D6); caution in hepatic impairment
Impact on Glucose Metabolism [21]	Neutral	May improve insulin sensitivity; favorable in diabetics	May worsen glycemic control	Improves insulin sensitivity; favorable in diabetics
Impact on Lipid Metabolism [22]	Neutral	May improve lipid profile	May adversely affect lipid profile	May improve lipid profile
Suitability in Diabetes [23], [24]	Suitable	Preferred due to metabolic benefits	Use with caution	Preferred due to metabolic benefits
Suitability in COPD/Asthma [25]	Preferred due to β1-selectivity	Use with caution; non-selective β-blockade may exacerbate symptoms	Use with caution; less β1-selective	Preferred due to β1-selectivity
Dosing Frequency	Once daily	Twice daily	Once daily	Once daily

Bisoprolol, with its low pharmacokinetic (PK) variability, offers more consistent drug exposure across individuals, reducing the need for frequent dose adjustments, making it a more reliable option than other BBs [26]. However, dose adjustment may be needed in elderly patients, as discontinuation is more common than in large mortality trials, primarily due to symptomatic hypotension limiting dose escalation [27]. In patients with severe renal or hepatic dysfunction, slow titration and close monitoring are advised due to extended half-life and accumulation potential [28].

Use of BBs in the management of HFrEF

In HFrEF, chronic β-adrenergic receptor activation leads to excessive catecholamine stimulation, myocardial apoptosis and fibrosis. BBs are fundamental in treating HFrEF due to their ability to attenuate the action of catecholamines, thereby improving myocardial function and reducing cardiac sympathetic overdrive [29]. Furthermore, effective use of BBs can help enhance the symptoms of HFrEF, minimize hospital readmissions, and increase survival duration [29]. Both Indian and international guidelines support the use of BBs (bisoprolol, carvedilol, or metoprolol) as a first-line treatment for HFrEF [7,12,30,31] (Table 2). Table 3 summarizes key clinical trials evaluating the efficacy of different BBs in HFrEF, including bisoprolol, carvedilol, and metoprolol, highlighting their comparative impact on mortality, hospitalization, and overall outcomes. Collectively, these trials confirmed the efficacy of BB therapy in HFrEF patients. The MERIT-HF trial demonstrated the clinical benefits of metoprolol CR/XL (controlled release/ extended release) in HFrEF patients, reducing mortality significantly by 34% [32]. Similarly, the COPERNICUS trial showed a 35% mortality reduction with carvedilol in HFrEF patients [33]. The US Carvedilol trial reported a 65% relative risk reduction (RRR) in mortality and CV hospitalization with carvedilol [34]. Bisoprolol is a recommended evidence-based BBs in guidelines for the management of HFrEF [12,31,35,36] (Table 2) and has shown significant efficacy and safety in patients with HFrEF through several randomized controlled trials (CIBIS, CIBIS II, and CIBIS III) (Table 4). The initial Cardiac Insufficiency Bisoprolol Study (CIBIS) evaluated 641 patients with HFrEF, and bisoprolol was associated with 20% relative risk reduction in mortality compared to placebo (relative risk 0.80, CI: 0.56, 1.15; p=0.22); however the result did not reach statistical significance largely due to being underpowered, with a small sample size and lower-than-expected event rates. However, bisoprolol significantly improved the patient’s New York Heart Association (NYHA) class (p=0.04) [37]. The subsequent CIBIS II trial [38], which included a larger cohort of 2647 patients, was stopped early due to a significant mortality benefit with bisoprolol. Treatment with bisoprolol led to 34 % RRR in all-cause mortality (HR: 0.66;95% CI 0.54-0.81; p < 0.0001) and 44% RRR in sudden death ( HR: 0.56;95% CI 0.39-0.80; p = 0.0011) compared to placebo [38]. This survival benefit was observed across all tolerated dose levels, and withdrawal was associated with a significant increase in mortality rate in the bisoprolol group with a relative hazard (RH) of 2.13 (95% CI=1.43-3.17, p=0.0002) [39]. Although post-hoc analyses are exploratory and not sufficient to drive guideline-level recommendations, findings from post-hoc analyses of the CIBIS II trials indicated consistent benefits of bisoprolol across various patient subgroups, including patients with or without diabetes, with or without renal impairment, with or without severe HF (NYHA class IV) symptoms, and patients with age ≥71 years Vs. <71 [40]. The third trial, CIBIS III, evaluated the sequence of initiating HF therapy with either bisoprolol or enalapril, an angiotensin-converting enzyme inhibitor (ACEi). The trial observed no significant differences in clinical outcomes between the initiation of bisoprolol or ACEi and the addition of another drug. However, it highlighted the importance of dose optimization, demonstrating that the therapy initiated first was more likely to be titrated to at least half of the maximal dose [41]. A meta-analysis of the CIBIS I and CIBIS II trials also proved the effectiveness of bisoprolol in reducing overall death, cardiovascular death, and hospitalizations [42].

**Table 2:** Guideline recommendation for use of beta blockers for HFrEF COR: Class of recommendation; LOE: Level of evidence; BP: Blood pressure; HF: Heart failure, HFrEF: Heart failure with reduced ejection fraction, BB: Beta blocker, OD: Once daily, CV: Cardiovascular.

Guideline	Recommendation	COR	LOE
European Society of Cardiology (ESC) 2021 [31]	BB is recommended for patients with stable HFrEF to reduce the risk of HF hospitalization and death	I	A
American Heart Association (AHA)/ACC/Heart Failure Society of America (HFSA) 2022 [12]	In patients with HFrEF, with current or previous symptoms, use of 1 of the 3 BBs proven to reduce mortality (i.e. bisoprolol, carvedilol, sustained-release metoprolol succinate) is recommended to reduce mortality and hospitalizations.	I	A
	Start evidence-based BB at their initial dose; bisoprolol to start at 1.25 mg OD; consider increasing dose every 2 weeks until maximum tolerated or targeted dose (10 mg OD) is achieved.		
Delphi Survey- based Consensus from Asia-Pacific 2023 [29]	BB are recommended as first-line treatment in patients with HFrEF to reduce all-cause mortality, CV mortality, and HF-related hospitalization. Consistent benefits have been observed by age, sex, cause (i.e., ischemic vs. non-ischemic cardiomyopathy), degree of symptoms, and degree of systolic dysfunction. They also improved left ventricular function.		
India Consensus 2020 [43]	At discharge from hospital: BB reduces the risk of all-cause and CV mortality but increase the risk of bradycardia and hypotension; once BP is stable, BB can be safely administered. The dose should be carefully increased to reduce the HR to around 70 beats per minute		

**Table 3:** Summary of major beta blocker trials in HFrEF HR: Hazard ratio, RAAS: Renin-angiotensin-aldosterone system, RR: Relative risk, RRR: Relative risk reduction, SNS: Sympathetic nervous system,↓: Decrease or reduction,↑: Increase or elevation.

Trial & Drug	Patients (n)	Comparator	HR / RR / RRR (95% CI)	Key Findings
Bisoprolol – CIBIS I [37]	641	Placebo	HR 0.80 [0.56–1.15], p=0.21	No mortality benefit; ↓ HF events
Bisoprolol – CIBIS II [38]	2,647	Placebo	HR 0.66 [0.54–0.81], p<0.0001	↓ All-cause & sudden death (HR 0.56 [0.39–0.80], p=0.0011)
Bisoprolol – CIBIS III [41]	1,010	Enalapril-first	HR 0.94 [0.77–1.16]	No difference in death/hospitalization for ACEI vs BB-first
Metoprolol CR/XL – MERIT-HF [32]	3,991	Placebo	RR 0.66 [0.53–0.81], p=0.0062	↓ All-cause death; ↓ sudden death (RR 0.59), ↓ HF death (RR 0.51)
Carvedilol – US Trial [34]	1,094	Placebo	RRR 65% [39–80], p<0.001	↓ Mortality; ↓ CV hospitalization
Carvedilol – COPERNICUS [33]	2,289	Placebo	RRR 35% [19–48], p=0.0014	↓ All-cause death; ↓ death/hospitalization (RRR 24%)

**Table 4:** Clinical evidence of Bisoprolol in HFrEF CIBIS: Cardiac Insufficiency Bisoprolol Study, HFrEF: Heart Failure with reduced Ejection Fraction, NYHA: New York Heart Association, LVEF: Left Ventricular Ejection Fraction, CHF: Chronic Heart Failure, ACEi: Angiotensin Converting Enzyme inhibitor, ARB: Angiotensin Receptor Blocker, BB: β Blocker, QD: Once a day, BID: Two times a day, HR: Hazard ratio, (HR <1.0 indicates reduced risk over time in the treatment group compared to control). CI: Confidence Interval, CV: Cardiovascular.

Study	Population	Number of patients	Dosage of bisoprolol/ comparator	Mean follow-up	Primary end point results	Other results
CIBIS I (Placebo-controlled RCT ) [37]	N=641 symptomatic HFrEF patients (LVEF <40%) NYHA class III or IV,	Bisoprolol (n=320) or placebo (n=321)	1.25 mg daily and progressively increased to 5 mg daily	1.9 ± 0.1 years.	All-cause mortality was lower with bisoprolol (16.6%) vs placebo (20.9%), but not statistically significant (RR 0.80; 95% CI 0.56–1.15; p=0.22)	Bisoprolol significantly improved functional status, with fewer hospitalizations for cardiac decompensation (61 vs. 90; p<0.01) and more patients improving by ≥1 NYHA class (68 vs. 48; p=0.04)
CIBIS II [38]	N=2647 symptomatic HFrEF patients NYHA class III or IV, (LVEF ≤35%)	Bisoprolol (n=1327) or placebo (n=1320)	1.25 mg daily and progressively increased to 10 mg daily	1.3 years	All-cause mortality was significantly lower with bisoprolol than placebo ( [11·8%] vs [17·3%] deaths with a HR of 0·66 (95% CI 0·54–0·81, p<0·0001)	20% less all-cause hospital admission in bisoprolol group than on placebo (p=0·0006) Significant reduction in combined endpoint of CV death and admission to hospital for CV events ( p=0.0004) Significant reduction in sudden death by 44%
CIBIS III [41]	N=1010 HFrEF patients (LVEF ≤35%) not receiving ACEi, BB, or ARB	Bisoprolol (n=505) or enalapril (n=505)	Bisoprolol (target dose 10 mg QD) or enalapril (target dose 10 mg BID) for 6 months, followed by their combination for 6–24 months	1–2.5 years	All-cause mortality : bisoprolol- was noninferior to enalapril- treatment In intent-to treat group, (HR 0.94; 95% CI 0.77 to 1.16, p=0.019) per-protocol sample (HR 0.97; 95% CI 0.78 to 1.21; P=0.046)	Death: 65 vs 73 pts (HR 0.88, P=0.44) Hospitalizations: 151 vs 157 (HR 0.95, p=0.66) Worsening of CHF requiring hospitalization: 63 vs 51 (HR 1.25, p = 0.23)

**Figure 2:**
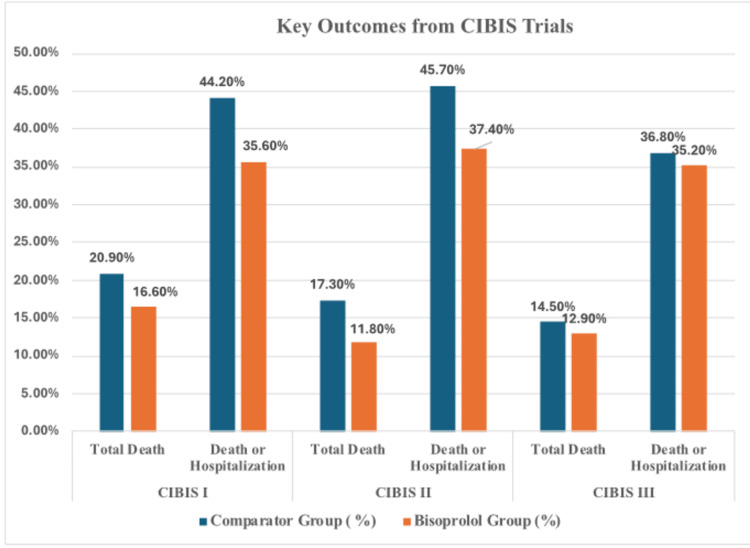
Key clinical outcomes from CIBIS trials comparing bisoprolol vs placebo (CIBIS, CIBIS II) and vs enalapril (CIBIS III) in HFrEF Reference numbers [37,38,41]. HFrEF: Heart failure with reduced ejection fraction.

Figure 2 provides a comparative visual summary of key outcomes, including total mortality and the composite endpoint of death or hospitalization, across the CIBIS I, II, and III trials. It highlights the consistent trend of improved outcomes in the bisoprolol groups versus comparator arms, establishing the clinical benefits of bisoprolol in patients with HFrEF.

Although BBs have comparable effectiveness in HFrEF management, pharmacokinetic differences, impact on glucose and lipid metabolism, and effects in the presence of comorbidities distinguish them from each other [26] (Table 1). The expert recommendations on the use of BBs in patients with HFrEF are given in Table 5. While bisoprolol remains a key component in the management of HFrEF, the role of fixed-dose combinations is increasingly recognized for their potential to enhance treatment adherence and simplify therapy. Looking ahead, combining bisoprolol with other pillars of GDMT, such as angiotensin receptor-neprilysin inhibitors (ARNI), sodium-glucose co-transporter-2 inhibitors (SGLT2i), and mineralocorticoid receptor antagonists (MRA)in a fixed-dose combination (FDC) format could represent a promising direction in HF management. Efforts are underway to explore the feasibility and potential clinical utility of some FDCs in HFrEF management.

**Table 5:** Table of recommendations

Experts' Opinion on Use of beta-blockers (BBs) in in HFrEF
BB is an essential component of GDMT for HFrEF, and its benefits are seen regardless of the dosage and sequence in the GDMT. BBs, including bisoprolol, should be initiated in patients with HFrEF unless contraindicated, starting at low doses and titrating up to target dose or the maximally tolerated dose. Bisoprolol is preferred BB considering high cardioselectivity, low pharmacokinetic variability, neutral effect on lipid levels and glucose metabolism making it suitable for use in patients who have cardiovascular risk factors, diabetes, or prediabetes.
Experts' Opinion on Use of BBs in Stable Angina Pectoris
BBs are to be used as the first-line therapy for the treatment of angina either as a single therapy or in conjunction with CCBs. Bisoprolol has shown long term effectiveness in angina patients. Bisoprolol is preferred for angina due to high cardioselectivity, low pharmacokinetic variability, neutral effect on lipid levels and glucose metabolism making it suitable for use in patients who have cardiovascular risk factors, diabetes, or prediabetes. Bisoprolol treatment is linked to better CV outcomes than other BBs and non-BB medications.
Experts' Opinion on Use of BBs in Patients with CVD having Renal Impairment
Compared to other BBs, bisoprolol is safer in patients with renal disease because of its favourable balance of pharmacokinetics, cardio selective action, minimal interactions, and tolerability. No adjustment of bisoprolol dosage required for patients with renal impairment. Cardioselective BBs like bisoprolol provide superior survival benefits than other BBs such as carvedilol among high-risk patients, like patients on maintenance haemodialysis.
Experts' Opinion on Use of BBs in Patients with CVD having Hepatic Impairment
Bisoprolol is safe to use in patients with liver impairments, however for hepatic cirrhosis and portal hypertension in end-stage cardiac failure, carvedilol is preferred.
Experts' Opinion on Use of BBs in Patients with CVD having Chronic Airway Diseases
Cardioselectivity of bisoprolol helps minimize respiratory side effects, making it safer for patients with chronic airway diseases like COPD. Bisoprolol is associated with a significantly better dose-dependent survival outcome in patients with HF and COPD compared to non-users. Bisoprolol is associated with better clinical outcomes in patients with COPD and CVD, including reduced all-cause mortality and lower rates of hospitalization for heart failure exacerbations compared to carvedilol. Bisoprolol leads to better lung function outcomes compared to other BBs such as carvedilol and metoprolol. Bisoprolol can be administered as an antianginal treatment in patients with moderate persistent bronchial asthma at a dose not exceeding 5 mg while avoiding bronchial obstruction.

Use of BBs in the management of stable angina pectoris

Various guidelines recommend the use of BBs as the first-line therapy for patients with angina (Table 5). The 2024 European Society of Cardiology (ESC) guidelines recommend BBs as the first-line treatment for angina either as a monotherapy or as a combination with calcium channel blockers (CCBs) (Class IB recommendation) [44]. The ESC guidelines appear to have a different stance on BB use compared to the American College of Cardiology/American Heart Association (ACC/AHA) guidelines (Table 5). This can be due to different approaches to guideline development and the interpretation of evidence.

Evidence from the literature also supports the effective use of bisoprolol in patients with stable angina (Table 6). Long-term (five years) data showed a significant reduction in mortality risk and improved cardiovascular outcomes in angina patients, supporting bisoprolol as a first-line therapy in real-world primary care over other BBs and non-BB agents [45]. Several studies have highlighted the efficacy of bisoprolol in reducing angina symptoms and improving exercise tolerance [46-48]. Studies demonstrating a comparison of the safety and efficacy of bisoprolol (10 mg once daily) with atenolol (100 mg once daily) reported a significant reduction in the weekly anginal attack rate, demonstrating equivalent effectiveness as an antianginal drug with no significant difference in nature and incidence of an adverse event [49-51]. Bisoprolol has also been compared with other anti-anginal medications, such as CCBs and nitrates. In a study by Van De Ven et al., bisoprolol was compared with isosorbide dinitrate and was found to be more effective at improving exercise tolerance during bicycle exercise testing and reducing the time to onset of angina, with fewer side effects [52]. In the Total Ischemic Burden Bisoprolol Study (TIBBS study) with 330 patients, bisoprolol showed significantly higher responder rates than nifedipine in reducing the number of ischemic episodes(100% reduction: 40.6% vs 14.8% at low dose; 52.5% vs 15.6% at high dose; p < 0.0001). Only bisoprolol showed a circadian effect by lowering the morning peak of ischemic episodes by 68% while with nifedipine, the circadian profile was unchanged [53]. Bisoprolol has also demonstrated long-term antianginal effects. Three-month therapy with bisoprolol in 6799 Stage 1 arterial hypertension patients showed a significant reduction in blood pressure (BP) and the number of angina attacks, with weekly episodes dropping from 2.03 to 0.91 [54]. In another study involving 222 hypertensive patients with stable angina, significant improvements were observed in flow-mediated vasodilatation (FMD) and myocardial survival after 12 months of treatment [55]. As reported by Terol et al., bisoprolol was well tolerated among 236 angina patients. Eighty-nine percent of patients reported a decrease in angina attacks; 56% were entirely free of attacks, and 5.1% showed beta blocker-specific side effects, illustrating the efficacy of bisoprolol and a favourable safety profile [56]. Expert recommendations on the use of BBs in patients with stable angina are presented in Table 7.

**Table 6:** Guideline recommendation for role of BBs in chronic stable angina pectoris COR: Class of Recommendation, LOE: Level of Evidence, Class I (strong recommendation), Class II (moderate recommendation), Level A: High-quality evidence from multiple randomized controlled trials (RCTs) or meta-analyses, Level B: Evidence from a single randomized controlled trial or large non-randomized studies, MI: Myocardial Infarction, LVEF: Left Ventricular Ejection Fraction.

Guideline	Patient profile	COR	LOE
American College of Cardiology /American Heart Association (ACC/AHA) 2012 [8]	Uncomplicated patient	I	B
	Previous MI	I	B
	Reduced LVEF (<40 %)	I	A
European Society of Cardiology (ESC) [57] 2024	Uncomplicated patient	I	B
	Previous MI	II	B
	Reduced LVEF (<40 %)	II	B
National Institute for Health and Care Excellence 2019 [58]	Uncomplicated patient	First line treatment (if symptoms are not controllable, switch to CCB or use both)	
	Previous MI		
	Reduced LVEF (<40 %)		
Canadian Cardiovascular Society 2014 [59]	Uncomplicated patient	II	B
	Previous MI	II	B
	Reduced LVEF (<40 %)	I	A

**Table 7:** Clinical evidence of bisoprolol instable angina pectoris BB: Beta blocker; HR: Heart rate; SBP: Systolic blood pressure; RPP: Rate-pressure product; GTN: Glyceryl trinitrate (nitroglycerin); CHD: Coronary heart disease; CAD: Coronary artery disease; TID: Three times a day; BID: Twice a day.

Study	Population	Sample size	Dosage of bisoprolol/ comparator	Findings
Sabido et al. 2019 [45]. Real-world study, cohort analysis	7607 angina patients	Bisoprolol (N=987) Other BBs (N=1348) Other than BBs (N=5272)	--	55% less mortality risk and 42% less risk of angina attack with bisoprolol vs other BBs
De Divitiis et al. 1987 [46]. Double-blind parallel groups	26 angina patients	Bisoprolol (N=13) Verapamil (N=13)	Bisoprolol 10-20 mg/day. Verapamil: 80-120 mg TID.	Both treatments significantly reduced angina episodes, nitroglycerin tablet consumption, ischemic episodes, and ST depression
Kato et al. 1986 [47]. Single blind study	36 stable angina patients	N=36	Bisoprolol 5 to 10 mg/day	Bisoprolol significantly reduced the HR frequency of anginal attacks, HR, SBP, and RPP at peak exercise and nitroglycerin consumption
Schnellbacher et al. 1986 [48]. Placebo-controlled double-blind crossover study	12 stable angina patients	N=12	Bisoprolol 10 mg/day. Atenolol 100 mg/day	Bisoprolol reduced exercise-induced symptoms, HR, RPP, and ischemic ST depression
MIRSA study [49]. Double-blind, randomized, parallel-group study	147 stable angina patients	Bisoprolol (N=76) Atenolol (N=71)	Bisoprolol 10 mg/day. Atenolol 100 mg/day	Both drugs significantly reduced angina attacks and RPP; similar exercise capacity improvement
Maltz et al. 1987 [50]. Double-blind, randomized, three-way crossover study	19 CAD and angina patients	N=19	Bisoprolol 5 mg/day, 10 mg/day. Atenolol 100 mg/day	All regimens reduced angina symptoms, GTN use, and HR (p<0.01); similar exercise time improvement
Kohli et al. 1986 [60]. Placebo-controlled double-blind RCT	20 stable angina patients	N=20	Bisoprolol 10 mg/day. Atenolol 100 mg/day	Bisoprolol and Atenolol improved exercise time and prolonged time to ST depression (p<0.001). A significant decrease in the peak exercise HR was seen with both drugs (p<0.001).
Van De Ven et al. 1995 [52] Double-blind, randomized crossover study	27 CAD and stable angina patients (Class II-III)	Treatment order group Nitrate/ Bisoprolol (N=14) Bisoprolol/nitrate (N=71)	Bisoprolol 10 mg/day. Isosorbide dinitrate 20 mg TID	Bisoprolol improved cardiac workload and time to angina onset; fewer side effects vs nitrates
TIBBS study 1995 [53]. Randomized double-blind, parallel group study	330 stable angina patients with transient ischemic episodes	Bisoprolol (N=161) Nifedipine SR (N=169)	Phase 1: bisoprolol (10 mg /day). Nifedipine SR (2 x 20 mg/day). Phase 2: bisoprolol (20 mg/day), nifedipine SR (2x 40 mg/day).	Statistically significant reduction in the number of transient ischemic episodes and total duration of ischemia (p<0.0001) by both drugs. Bisoprolol demonstrated a distinctive circadian impact by reducing the morning peak of transient ischemic episodes by 68% compared to Nifedipine SR. Bisoprolol lowered the HR significantly (p < 0.001), while nifedipine had no effect on HR

Use of BBs in patients with CVD and renal impairment

The prevalence of concomitant HF and chronic kidney disease (CKD) is high, with 40% of patients affected by HF demonstrating renal dysfunction. As well as, the proportion of HF in CKD patients ranges between 17-50% depending on the stage of CKD and the patient’s age [61]. Studies have described the clinical advantages of BB therapy irrespective of CKD stage, and indicated significant reductions in all-cause mortality and cardiovascular outcomes in patients with HF [62,63]. Bisoprolol is mainly metabolized in the kidneys as well as the liver, since both organs contribute to half of the elimination of the drug each. This balanced excretion reduces the dependency on any single organ for drug elimination, minimizing the risk of drug accumulation and toxicity in patients with either renal or hepatic impairment. In addition, bisoprolol plasma level remains consistent, which reduces the need for frequent dose adjustment in patients with mild-to-moderate dysfunction. However, bisoprolol dosage should not exceed 10 mg daily in severe and end-stage renal failure [64]. In contrast, the BBs, such as atenolol and metoprolol, depend greatly on renal and hepatic elimination, respectively, which may cause adverse effects in patients with impairment of one of the elimination organs [64]. Unlike metoprolol and carvedilol, which are primarily metabolized by the liver through the cytochrome P450 system, especially CYP2D6, bisoprolol undergoes minimal CYP450 metabolism. This reduces its potential for drug-drug interactions, which is particularly important in patients with renal impairment on multiple medications [65].

Another factor contributing to the safety profile of BBs is dialyzability, as this significantly influences clinical decision-making in patients undergoing hemodialysis, affecting drug clearance, dosing strategies, and therapeutic consistency. Highly dialyzable BBs (e.g., metoprolol, atenolol) are substantially removed during dialysis, which may lead to subtherapeutic levels post-session, reducing cardiovascular protection and requiring additional dosing adjustments. Although bisoprolol is poorly dialyzable, it has been shown to be partially cleared during hemodialysis, but to a significantly lesser extent than atenolol or metoprolol. However, it maintains more stable plasma levels due to its high bioavailability and balanced renal and hepatic clearance, making it a safer and more consistent β-blocker option for hemodialysis patients [66].

In contrast, carvedilol, though poorly dialyzable, with additional α-1 blocking properties, is more likely to cause intradialytic hypotension in dialysis patients [67,68]. In patients with severe renal impairment (creatinine clearance <10 mL/min), bisoprolol exposure doubles, and its plasma elimination half-life extends to approximately 24.2 hours [28]. The subgroup analysis of the CIBIS II trial also demonstrated the efficacy of bisoprolol in HFrEF patients with renal impairment. Mortality risk was similarly reduced in patients with CrCl ≤ 60 mL/min (RR 0.66, 95% CI 0.50-0.88) and >60 mL/min (RR 0.69, 95% CI 0.54-0.89), with no significant interaction (p=0.69) [40]. In dialysis-dependent patients, cardioselective BBs are associated with 15% fewer CV events (HR =0.85; 95% CI =0.81, 0.89) and 17% lower all-cause mortality (HR =0.83; 95% CI =0.69, 0.99) than non-selective BBs [69].

Furthermore observational data suggest that, bisoprolol was associated with lower all-cause mortality (HR 0.66; 95% CI 0.60-0.73) and reduced risk of major adverse cardiovascular events (MACEs) (HR 0.85; 95% CI 0.80-0.91), HF (HR 0.83; 95% CI 0.77-0.92), and ischemic stroke (HR 0.84; 95% CI 0.72-0.97) compared to carvedilol in patients undergoing maintenance hemodialysis for more than 90 days [67,70]. Robust real-world observational data can help guide BB choice in patients undergoing dialysis, but should be interpreted cautiously due to potential confounding factors, bias, and patient variability.

Use of BB in patients with CVD and hepatic impairments

Hepatic dysfunction is a common comorbidity in patients with CHF and CVD, often complicating pharmacologic management due to altered drug metabolism and increased vulnerability to adverse effects. Liver impairment is prevalent in CHF [71], and choosing an appropriate BB can be challenging in this population. Among BBs, carvedilol, a non-selective BB, is the drug of choice for patients with cirrhosis. Carvedilol has been shown to offer a significant survival advantage, with a median survival of 7.8 years [72], and achieves a higher rate of hemodynamic response in reducing portal pressure, a major complication of cirrhosis, compared to propranolol, further supporting its potency in cirrhotic patients with portal hypertension [73].

However, when considering overall cardiovascular protection in patients with cirrhosis beyond portal hypertension, a 13-year nationwide population-based study in Asia found that compared to nonselective β1-blockers, β1-selective blockers (bisoprolol, metoprolol, atenolol) were associated with a 38% lower risks of major adverse cardiac and cerebrovascular events (HR=0.62; 95% CI =0.42-0.91; p=0.014, a 34% reduction in all-cause mortality (HR=0.66; 95% CI= 0.38-1.14; p=0.135, and a similar non-significant trend was observed for worsening liver outcomes in cirrhosis patients (HR=0.66; 95% CI=0.38-1.14; p=0.354) [74].

Use of BBs in patients with CVD with glucose and lipid abnormalities

Bisoprolol has been described to have a generally neutral effect on lipid metabolism, which makes it the preferred BB for patients with vulnerable lipid profiles. Unlike non-selective BBs, which can raise triglycerides and LDL cholesterol while lowering HDL cholesterol, bisoprolol is less likely to cause metabolic derangements that include lipid levels, insulin sensitivity, and alterations in blood sugar than non-selective and partially selective BBs [75]. Bisoprolol did not adversely affect lipid profiles even in the presence of cardiovascular risk factors and type 2 diabetes [76]. A comparison of bisoprolol’s effects on lipid profile and glucose metabolism versus other BBs is presented in Table 1.

In the TENACITY study, bisoprolol improved left ventricular ejection fraction (LVEF) and reduced HR in post-MI patients with left ventricular dysfunction, without adversely altering lipid or glucose metabolism [13]. In a meta-analysis involving bisoprolol versus other BBs, including metoprolol and atenolol, bisoprolol demonstrated its effectiveness in lowering BP and heart rate with significant improvement in HDL cholesterol at 52 weeks (p<0.01) and 104 weeks (p<0.01) [77].

Another positive aspect of bisoprolol is its neutrality or even the possibility of a slightly beneficial effect on glucose metabolism, while being used in patients with HF and angina having diabetes or prediabetes [78]. A double-blind, crossover study indicated that bisoprolol 10 mg OD did not affect blood glucose and HbA1C in patients with diabetes compared to placebo, confirming its safety profile in this population [79]. A similar neutral effect was observed in young individuals with hypertension and obesity [76].

Use of BBs in patients with CVD and chronic airway diseases

Chronic obstructive pulmonary disease (COPD) and asthma are often comorbid with CVDs such as ischemic heart disease, congestive heart failure (CHF), and cardiac arrhythmia. Similarly, 10-46% COPD patients show evidence of abnormal LVEF [80]. Despite their clinical benefits, BBs are often under-prescribed in this population because of concerns over bronchospasm. However, emerging evidence supports the safe and effective use of cardioselective BBs in patients with chronic airway diseases and coexisting CVD.

COPD

A meta-analysis evaluating the use of BBs in COPD patients with concomitant CVD has documented clinical benefit in improving survival and decreasing the risk of re-hospitalization [81,82]. Neef et al. [83] showed that cardioselective β1-blockers are well tolerated in COPD patients, even during hospitalization for exacerbation.

As being cardioselective BBs, bisoprolol selectively blocks β-1 adrenergic receptors primarily found in the heart, with less impact on β-2 receptors in the lungs. This selectivity is crucial for minimizing respiratory side effects in patients with chronic airway diseases [84]. It is associated with improved clinical outcomes in patients with COPD and CVD compared to non-selective BBs such as carvedilol and metoprolol. Its use in patients with COPD and HF has been linked to reduced all-cause mortality and lower rates of hospitalization due to HF exacerbations [85-87]. A study by Su et al. involving 11,558 patients found that bisoprolol use was associated with a significant dose-dependent mortality benefit, low-dose HR, 0.76 (95% CI, 0.59-0.97, p = 0.030), high-dose bisoprolol HR, 0.40 (95% CI, 0.26-0.63), p< 0.001) compared to non-users [85]. Similar improved clinical outcomes have been reported in other studies [86,87]. These outcomes include a lower incidence of CHF and/or COPD exacerbation (p=0.033) [87], reduced mortality, and decreased hospitalisation rate [71] in the bisoprolol arm compared to the carvedilol group.

A comparison of bisoprolol and carvedilol in 63 BB naive patients with CHF and moderate-to-severe COPD found a significant increase in forced expiratory volume in one second (FEV1) in the bisoprolol arm (1.56 ± 0.41 to 1.70 ± 0.52 L; p=0.046) but not in the carvedilol arm [88]. In a triple-crossover trial, bisoprolol led to the highest FEV1 among the beta blockers tested, followed by metoprolol and carvedilol [89].

Additionally bisoprolol reduced mortality and hospitalization rate due to CHF exacerbations more effectively than carvedilol and metoprolol in patients with coexistent CHF and COPD (mortality: adjusted hazard ratio (aHR) =0.51, 95% CI, 0.29-0.89; hospitalization rate due to CHF exacerbation: aHR =0.48, 95% CI, 0.23-1.00) [86]. A systematic review and meta-analysis involving 3269 adults with COPD with or without HF who were treated with bisoprolol concluded that compared to the control group, bisoprolol improved lung function and exercise performance while reducing inflammatory markers without significant adverse effects. Bisoprolol showed improvements in FEV1 (mean difference (MD) 0.46, 95% CI 0.27 to 0.65, p=0.000), FEV1% (MD 0.64, 95% CI 0.42 to 0.86, p=0.000), and a modest increase in FVC (MD 0.20, 95% CI 0.05 to 0.34, P=0.008) [90]. In patients with non-asthmatic chronic obstructive lung disease and co-existing stable angina pectoris, bisoprolol was found to be effective in its therapeutic dose range (5-20 mg OD), with no increased wheeziness or shortness of breath, highlighting its safety and efficacy in such patients [91].

Asthma

The use of cardioselective BB in patients with asthma and CVD did not significantly increase the risk of moderate or severe asthma exacerbations. A meta-analysis of 19 trials evaluating the effects of cardioselective ß-blockers on FEV1 found that a single dose led to a 7.46% decrease in FEV1 [92]. In a network meta-analysis, assessing risk of exacerbation with various BB in asthma patients, oral bisoprolol had a risk ratio (RR) of 0.46 (95% CI 0.02-11.65), suggesting that bisoprolol does not significantly increase the risk of asthma attacks [93]. However, the extremely wide confidence interval reflects substantial statistical uncertainty, limiting the ability to draw definitive conclusions. It can be administered as an antianginal treatment in patients with moderate persistent bronchial asthma at a dose not exceeding 5 mg to prevent bronchial obstruction [94]. The expert recommendations on the use of BBs in patients with chronic airway diseases are presented in Table 7.

Contraindications for bisoprolol

Bisoprolol is contraindicated in patients with marked sinus bradycardia, cardiogenic shock, and complete heart block, and should be used with caution in those with second-degree AV block or recent fluid retention without a diuretic. They are generally safe in mild to moderate asthma or COPD, but should be avoided in severe cases. In diabetes, they may mask hypoglycemia, requiring close monitoring.

Study limitations

This expert opinion provides valuable, practice-oriented guidance on the use of bisoprolol in HFrEF and stable angina, with both clinical trial evidence and real-world experience. However, its generalizability is limited by the lack of direct head-to-head comparisons with other BBs, limited representation of specific patient populations, such as the elderly, those with CKD, and COPD, and reliance on expert consensus rather than prospective, large-scale studies.

## Conclusions

Bisoprolol, as one of the selective β1-adrenergic antagonists, plays a crucial role in the treatment of HFrEF and stable angina pectoris. Its β1 selectivity and balanced hepato-renal metabolism provide a reliable and predictable therapeutic effect, which makes it suitable for patients with HFrEF and stable angina having renal or hepatic impairment, and COPD. While bisoprolol has shown favorable outcomes in patients with COPD, careful patient selection remains essential when used in those with chronic airway diseases, particularly in asthma or individuals with severe airway reactivity, where safety data are limited.

Literature evidence and opinions of experts confirm that bisoprolol is effective in lowering mortality, hospitalization, and major cardiovascular events risk. In addition, it has minimal intrinsic sympathomimetic activity and high cardio-selectivity, which affords it safety over the long-term in patients with CVDs. These characteristics make bisoprolol a preferred BB in both HFrEF and angina in routine clinical practice scenarios. While current evidence is encouraging, more large-scale, prospective comparative studies are warranted to further validate bisoprolol’s role across diverse patient subgroups. Moreover, while bisoprolol remains a key component in the management of HFrEF, its integration with other pillars of GDMT through FDC offers a promising strategy to improve treatment adherence and streamline therapy. Further exploration and clinical evaluation of such combinations may help optimize patient outcomes.
